# RNAa-mediated overexpression of WT1 induces apoptosis in HepG2 cells

**DOI:** 10.1186/1477-7819-10-11

**Published:** 2012-01-13

**Authors:** Qi Qin, Yi-Wei Lin, Xiang-Yi Zheng, Hong Chen, Qi-Qi Mao, Kai Yang, Shou-Jiang Huang, Zheng-Yan Zhao

**Affiliations:** 1 Department of General Surgery, Children Hospital, Zhejiang University School of Medicine, Hangzhou 310006, China; 2 Department of Urology, the First Affiliated Hospital, Zhejiang University School of Medicine, Hangzhou, 310003, China

**Keywords:** WT1, Small activating RNA, dsRNA, Hepatocellular carcinoma, HepG2 cell, Apoptosis

## Abstract

**Aim:**

Recent studies have reported that double-stranded RNA (dsRNA) can activate gene expression by targeting promoter sequence in a process termed RNA activation. The present study was conducted to evaluate the potential of WT1 induction by small activating RNA targeting the WT1 promoter (dsWT1) in the treatment of hepatocellular carcinoma.

**Methods:**

The human hepatocellular carcinoma cell line HepG2 was transfected with dsRNA by liposomes. The expression of mRNA and protein in cells were investigated using real-time reverse real-time quantitative PCR and Western blot, respectively. Cell viability and clonogenicity were determined by MTT assay and clonogenicity assay, respectively. Cell apoptosis was evaluated by flow-cytometric analysis.

**Results:**

Expressions of WT1 mRNA and protein in dsWT1 treated HepG2 cells were significantly elevated. Inhibition of cell viability by dsWT1 was dose-dependent and time-dependent. Reduction of the number and size of colonies formed were found in dsWT1 treated cells. dsWT1 induced significant apoptosis in HepG2 cells. The decreased anti-apoptotic protein Bcl-2 and elevated pro-apoptotic protein Bak expression were detected in dsWT1 treated cells. The level of pro-caspase-3 remarkably decreased and cleaved caspase-3 and PARP fragment were also detected in dsWT1 treated cells.

**Conclusion:**

These data show that RNAa-mediated overexpression of WT1 may have therapeutic potential in the treatment of hepatocellular carcinoma.

## Background

Hepatocellular carcinoma (HCC) is one of the most common malignancies in the world, and the prognosis of patients with HCC is very poor [1]. As it is geographically biased toward the several parts of Asia and Africa, China in particular, it presents one of the major health threat in China [2]. Although several treatments such as tumor resection, liver transplantation, transcatheter arterial chemoembolization (TAE), and local radiofrequencyablation (RFA) are now used to treat HCC, there is no overall long-term survival benefit so far [3]. Therefore, strategies that explore new therapy for HCC are urgently needed.

Recently, Li, et al. and others have reported that double-stranded RNA (dsRNA) can activate gene expression by targeting promoter sequence in a process termed RNA activation [4,5]. This technique alters chromatin structure leading to robust and prolonged expression of the endogenous target gene [4]. As such, RNAa has potential to be a useful tool for interrogating gene function by serving as an alternative to traditional vector-based systems and an attractive strategy to activate tumor suppressor genes for the treatment of cancer [6].

Wilms' tumor 1 gene (WT1) is an important nuclear factor involved in organ development and cell growth [7]. The role of WT1 in cell biology is equally complex, and it has been shown that the repression or activation function of WT1 is dependent on the cell type and on its level of expression [8]. Moreover, WT1 has been described as a tumor suppressor and as an oncogene [9]. It was reported that plasmid-mediated transfection of WT1-KTS isoforms into hepatoma cell lines induced p53-independent apoptosis [10]. Recently, some studies showed WT1 is expressed in several human hepatocellular carcinoma (HCC) cell lines, and is also expressed in tumor tissue in 42% of patients with HCC [11]. However, the role of WT1 in hepatocarcinogenesis has not been clarified.

In this study, we investigate the effects of the dsRNA that specifically targets the promoter region of WT1 on the growth of human hepatocellular carcinoma cells HepG2. We found that the dsRNA that specifically targets the promoter region of WT1 could up-regulate WT1 and induce apoptosis which was related to modulation of Bcl-2 family.

## Materials and methods

### Reagents

The sequence of dsRNAs (dsWT1-319: S,5'-GAC UCA CUG CUU ACC UGA A[dT][dT]-3';AS,5'-UUC AGG UAA GCA GUG AGU C[dT][dT]-3') and dsControl: S, 5'-ACU ACU GAG UGA CAG UAG A[dT][dT]-3';AS, 5'-UCU ACU GUC ACU CAG UAG U[dT][dT]- 3') were designed as previously reported [12] and chemically synthesized by GeneChem (Shanghai, China). Primary immunoblotting antibodies were: anti-WT1, anti-Bcl-2, anti-Bak and anti-poly (ADP-ribose) polymerase (PARP) (Santa-Cruz Biotechnology, Inc., Santa Cruz, CA), anti-β-actin (Cell Signaling Technology, Beverly, MA).

### Cell culture and transfection

The human hepatocellular carcinoma cell line HepG2 was obtained from the Shanghai Institute of Cell Biology, Chinese Academy of Sciences. The cells were cultured in RPMI 1640 medium supplemented with 10% heat-inactivated fetal bovine serum, penicillin (100 U/mL), and streptomycin (100 mg/L) in a humidified atmosphere containing 5% CO_2 _maintained at 37°C. The day before transfection cells were plated in growth medium without antibiotics at a density of 30-40%. Transfections of dsRNA were carried out by using Lipofectamine 2000 (Invitrogen, Carlsbad, CA) according to the manufacturer's protocol and lasted for 24, 48 or 72 h. Cell images were taken using a phase-contrast microscope at 100× magnification (Olympus, Japan).

### Cell viability assay

Cell viability was determined by the MTT assay. Approximately 2,000 HepG2 cells were plated in each well of a 96-well plate. After overnight incubation, the cells were treated with dsRNAs for 48-72 h and the concentration of dsWT1-319 arranged from 2 to 50 nM. At the various times following treatment, the medium was removed and MTT (20 μl of 5 mg/mL) was added to each well and incubated at 37°C for 4 h. The plates were spun, and the purple colored precipitates of formazan were dissolved in 150 μl of dimethyl sulfoxide. Absorbance was measured at 490 nm using the MRX II absorbance reader (DYNEX Technologies, Chantilly, Virginia, USA). The reduction in viability of in dsWT1 or dsControl treated HepG2 cells were expressed as a percentage compared to mock cells. Mock cells were considered to be 100% viable.

### Colony formation assay

Exponentially growing cells were plated at approximately 2,000 cells per well in 6-well plates and transfected with dsRNA. Culture medium was changed every 3 days. Colony formation was analyzed 12 days following transfection by staining cells with 0.05% crystal violet solution for 1 hour.

### Real-time quantitative PCR (qPCR)

Total RNA was extracted from cells transfected for 48 h (mock, 50 nM dsControl, 50 nM dsWT1-319) and reverse transcribed using random primers. The resulting cDNA was quantified by the SYBR Premix Ex Taq™ Kit (Takara, Dalian, China) according to the manufacturer's protocol in a ABI Prism 7500 Real-time PCR detection system (Applied Biosystems, CA). GAPDH mRNA levels were used for normalization. Values are expressed as fold-difference compared to mock. Primer sequences forWT1 are 5'- AGAGCCAGCCCGCTATTC-3' (forward) and 5'- GGCGTCCTCAGCAGCAAA-3' (reverse) and, for GAPDH are 5'- AAGGTGAAGGTCGGAGTCA-3' (forward) and 5'- GGAAGATGGTGATGGGATTT -3' (reverse).

### Detection of apoptotic cells by flow cytometry

A quantitative assessment of apoptosis was made by determining the percentage of cells with nuclei that were highly condensed or fragmented. Cells were harvested at 48 or 72 h following dsRNAs treatment (mock, 50 nM dsControl, 50 nM dsWT1-319) as described above, and washed twice with pre-chilled PBS and resuspended in 100 μL binding buffer at a concentration of 1 × 10^6 ^cells/mL. Annexin V and PI double-staining was performed using the Annexin V-FITC Apoptosis Detection Kit (BD Biosciences, San Jose, CA, USA) as described by the manufacturer's protocol. Cell apoptosis analysis was performed by Beckman Coulter FC500 Flow Cytometry System with CXP Software (Beckman Coulter, Fullerton, CA, USA) within 1 h.

### Western blotting analysis

Briefly, at 72 h following dsRNA treatment, cells were harvested, washed, and lysed with lysis buffer as described above. Protein concentration in the resulting lysate was determined using the bicinchoninic acid protein assay kit (Pierce Biotechnology, Rockford, IL, USA) according to the manufacturer's instructions. Equivalent quantities of protein (30-50 μg) were separated by electrophoresis in 8% Tris-glycine polyacrylamide gels and transferred to nitrocellulose membranes. Membranes were blocked and then incubated overnight with the appropriate primary antibody at dilutions specified by the manufacturer. They were next washed three times in 15 mL TBS-Tween and incubated with the corresponding horseradish peroxidase (HRP)-conjugated secondary antibody at 1:2,000 dilution in TBS-Tween for 2 h. Bound secondary antibody was detected using an enhanced chemiluminescence (ECL) system (Pierce Biotechnology).

### Statistical analysis

All values were expressed as mean ± SD. Statistical significance was compared between treatment group and controls using one-way analysis of variance (ANOVA). Data were considered statistically significant when p values were < .05.

## Results

### dsWT1-319 induces WT1 gene expression in HepG2 cell line

**Figure 1:**
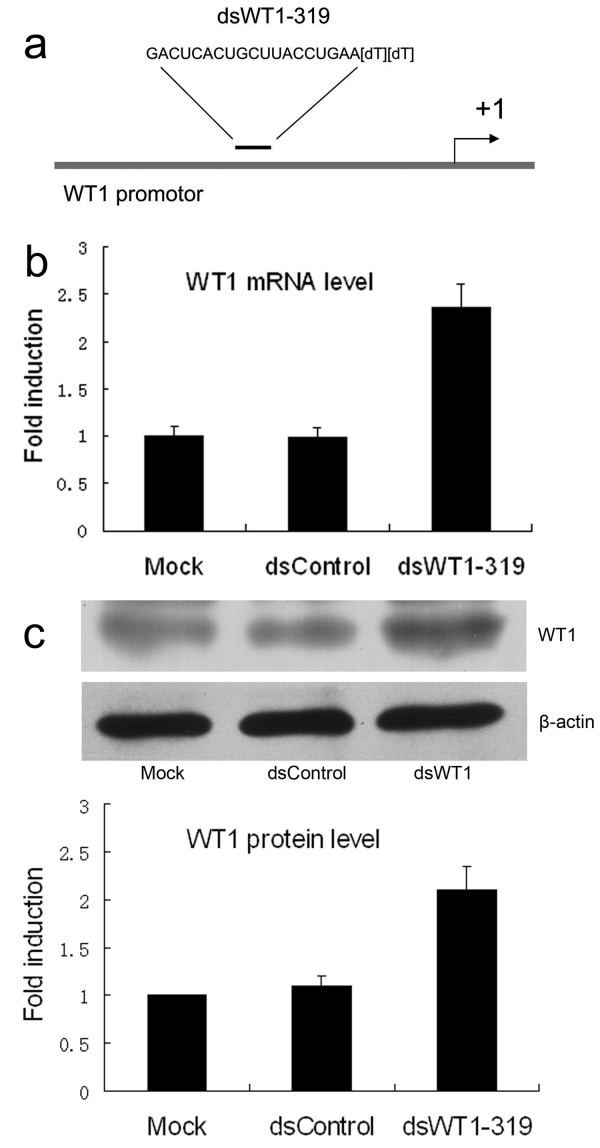
**dsWT1-319 induces WT1 gene expression in HepG2 cell line Cells were transfected with 50 nM dsRNA for 48 h**. (a) A schematic representation of the WT1 promoter and the location of the dsRNA target. (b) Induction of WT1 mRNA expression was detected by qPCR. The results were normalized to GAPDH and presented as means ± SD of three independent experiments.(Mock:1.00 ± 0.11, dsControl:0.99 ± 0.10, dsWT1-319:2.37 ± 0.24). (c) Induction of WT1 protein expression was detected by Western blot analysis. β-actin levels were also detected and served as a loading control. The WT1 protein expression levels were normalized to β-actin and the results are presented as means ± SD of three independent experiments. (Mock:1.00 ± 0.01, dsControl:1.10 ± 0.10, dsWT1-319:2.10 ± 0.25)

A dsRNA targeting the WT1 gene promoter at position-319 relative to the transcription start site (dsWT1-319) was used to activate WT1 expression (Figure 1). HepG2 cells were transfected with 50 nM of dsWT1-319 and a control dsRNA (dsControl). Forty-eight hours later, expression of WT1 mRNA and protein was detected by qPCR and Western blotting analysis, respectively. Expression of WT1 in dsWT1-319 treated cells was significantly elevated. Compared to mock and dsControl transfections, dsWT1-319 caused an over 2-fold induction in both mRNA and protein level (Figure 1).

### dsWT1-319 inhibits HepG2 cell growth, viability and clonogenicity

**Figure 2:**
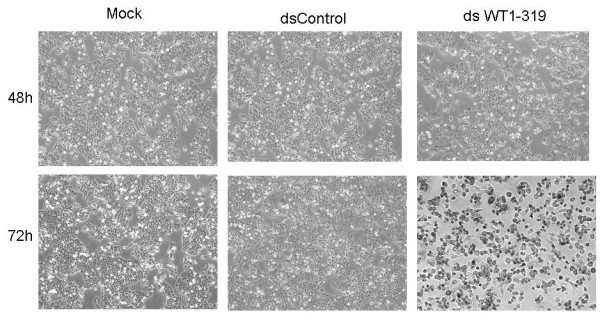
**dsWT1-319 targeting the WT1 promoter inhibited HepG2 cell growth**. HepG2 cells were transfected with 50 nM dsWT1-319, 50 nM dsControl or mock. Cell images were taken at 48 and 72 h after transfection at 100× magnification. dsWT1-319 transfected cells were less dense and more dead cells were observed than dsControl and mock transfections.

The dsWT1-319 and dsControl were transfected into HepG2 cells at the concentration of 50 nM. At 48 h and 72 h following transfection, phase-contrast images of cells from the same fields were taken. Morphologically, mock and dsControl transfected cells maintained healthy growth after transfection, whereas cells transfected with WT1 dsRNA gradually lost viability and the number were evidently less after 72 h (Figure 2).

**Figure 3:**
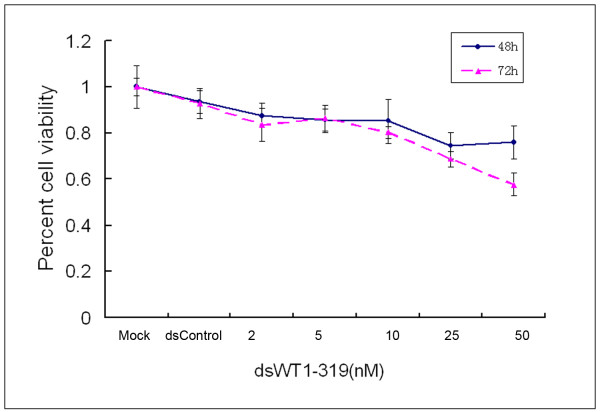
**dsWT1-319 inhibited cell viability of HepG2 cells in a dose-dependent and time-dependent manner by the MTT assay**. Reduced cell viability was observed with dsWT1-319 treatment (2-50 nM) at 48 and 72 h. The data are presented as means ± SD (n = 8).

**Figure 4:**
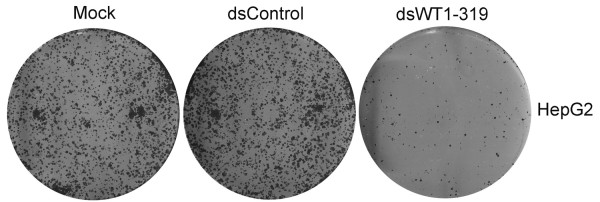
**HepG2 cells were plated at 1,000 cells per well in 6-well tissue culture plates and transfected with mock, dsControl, or dsWT1-319**. Cells were grown for 12 days and analyzed for colony formation by staining with crystal violet. Shown are representative photographs taken of tissue culture plates from each dsRNA treatment group following staining for colony formation.

The effect of dsWT1-319 on proliferation and viability of HepG2 cells was determined with varying concentrations of dsWT1-319 and times (48-72 h) by MTT assay. As shown in Figure 3, the effects of dsWT1-319 on cell viability occurred within 48 h and at dsRNA concentrations as low as 2 nM. Inhibition of cell viability by dsWT1-319 (10-50 nM)was both dose- and time-dependent. Cell viability with dsRNA treatment at concentrations of 2-50 nM after 48 h ranged from 87.7% to 76.0%, whereas after 72 h ranged from 83.6% to 57.8% (Figure 3). Clonogenicity assay revealed the reduction of the number and size of colonies formed in dsWT1-319 treated cells(Figure 4).

### dsWT1-319 induces significant apoptosis in HepG2 cells

**Figure 5:**
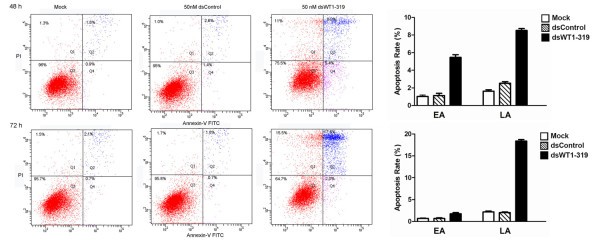
**dsWT1-319 treatment induced time-dependent apoptosis in HepG2 cells detected by flow cytometry using a double-staining method with FITC-conjugated annexin V and PI**. Annexin V-stained cells indicates the early apoptotic cells, whereas Annexin V + propidium iodide-stained cells are the late apoptotic cells. A representative blot is shown from three independent experiments with identical results.

The dsWT1-319 mediated loss of HepG2 cell viability and apoptosis were evaluated by flow-cytometric analysis of dsRNA-treated cells labeled with PI and Annexin V. As shown in Figure 5, we found that dsWT1-319 caused a time-dependent increase in HepG2 cell apoptosis. The number of early apoptotic cell at 48 h (5.5 ± 0.7% *vs *1.2 ± 0.3%) and 72 h (2.1 ± 0.4% *vs *0.9 ± 0.3%) following dsWT1-319 treatment increased significantly as compared with control treatments(*P *< 0.05), and number of late apoptotic cell at 48 h(8.3 ± 1.1% *vs *2.2 ± 0.4) and 72 h(17.9 ± 2.3 *vs *2.1 ± 0.3) following dsWT1-319 treatment also increased significantly as compared with control treatments(*P *< 0.01) These data also showed that dsWT1-319 treatment resulted in cell necrosis(15.4 ± 1.7%) in cells treated for 72 h, which might be a secondary event in the apoptotic process.

### The relationship of dsWT1-319 treatment with the expression of apoptosis related proteins

**Figure 6:**
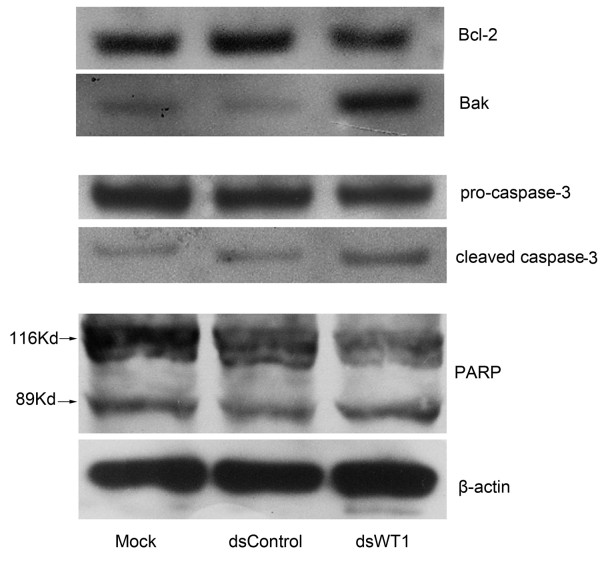
**The expressions of apoptosis-related proteins in treated cells were analyzed by Western blotting**. β-actin levels were also detected and served as a loading control. A representative blot is shown from three independent experiments with identical results. Fifty nanomolars dsWT1-319 treatment reduced the level of Bcl-2, increased the level of Bak, and activated caspase-3 and PARP in HepG2 cells at 72 h following treatment.

Bcl-2 is known as an anti-apoptotic protein and Bak as an proapoptotic protein, so we detected their expression after 50 nM dsWT1-319 treatment for 72 h. Consistent with the significantly increased HepG2 apoptosis, the level of Bcl-2 was decreased and level of Bak were elevated in dsWT1-319 treated cells compared to mock and dsControl treated ones (Figure 6). Caspase-3 and poly (ADP-ribose) polymerase (PARP) play central roles in apoptosis. We observed that the level of pro-caspase-3 remarkably decreased in 50 nM dsWT1-319 treated cells at 72 h following treatment. The cleaved caspase-3 and 89 kDa cleaved PARP fragment were detected in dsWT1-319-treated samples(Figure 6). Thus the significant changes of apoptosis- related proteins caused by dsWT1-319 confirmed the observed apoptosis above and the anti-tumor effect of dsWT1-319 on HepG2 cells.

## Discussion

RNA activation (RNAa) is a newly discovered mechanism of gene activation directed by small double-stranded RNAs (dsRNAs) [4,5,12,13]. It offers similar benefits as RNAi by utilizing small dsRNAs, while representing a new method for gene overexpression [12]. Several models of RNAa have been reported or proposed including transcriptional activation by targeting promoter-specific sequences [4,12,14] and/or gene antisense transcripts [15,16] leading to changes in chromatin structure at the targeted gene. RNAa is generally potent and long-lasting making it a promising therapeutic strategy for diseases that can be corrected by stimulating gene expression [4,12]. Vector-based overexpression is the traditional approach to evaluate the function of tumor suppressor genes or oncogenes in cancer cells. However, all vector-based systems require ectopic expression from an exogenous construct. Ectopic expression vectors do not typically resemble natural genes [17]. Ideally, RNAa can be applied as a cancer treatment to re-activate tumor suppressor or pro-apoptotic genes that are otherwise not targetable by current therapeutic strategies [12].

WT1 was initially discovered as a tumor suppressor in Wilms' tumor (WT), a pediatric kidney malignancy that affects approximately 1/10,000 children. The Wilms' tumor suppressor protein WT1 functions as a transcriptional regulator of genes controlling growth, apoptosis, and differentiation [18]. Recent findings have shown that wildtype WT1 is expressed in a variety of tumors from different origins that normally do not express WT1 [7]. Several reports have revealed an antiapoptotic function for WT1, suggesting that WT1 acts as an oncogene in some tumors [18]. However, the ability of WT1 to induce growth suppression and suppress tumorigenicity in mice also highlights its role as a tumor suppressor. For example, The stable introduction of the WT1 -/- isoform into G401, a kidney-derived tumor cell line that does not express endogenous WT1, alters cellular morphology and reduces tumor formation in athymic nude mice [19]. Furthermore, expression of WT1 in osteosarcoma cell lines, Saos-2 and U20S can alter signaling pathways and induce apoptosis [20], and plasmid-mediated transfection of WT1-KTS isoforms into in HCC cell lines, Hep3B and HepG2 also induced apoptosis [10].

Recently, Li et al [12] has confirmed that dsWT1-319 can up-regulate expression of WT1 in both African green monkey (COS1) and chimpanzee (WES) cells. In this study, we focus on investigating the effects and efficacy of WT1 induction by small dsRNA in the treatment of hepatocellular carcinoma. We found that dsWT1-319 induced activation of WT1 inhibited cell viability in a dose- dependent and time-dependent way by MTT assay and it was related to apoptotic cell death after treatment. The role of WT1 in hepatocarcinogenesis has not been clarified. It is reported that WT1 is expressed in several human hepatocellular carcinoma (HCC) cell lines, including PLC/PRF/5 and HepG2, and in HCC tumor tissue in a high proportion of patients, up-regulation of WT1 in liver cells promotes apoptosis resistance and cellular dedifferentiation. Moreover, overexpressed WT1 was associated with a poor prognosis of HCC [11,21]. The mechanism of WT1 in the regulation of apoptosis remains unclear, several genes that are central to the control of apoptosis have been proposed as targets of WT1, including Bcl-2, Bcl-2A1, Bak, c-myc, and JunB [18,22]. Also, WT1 can downregulate growth factor receptors such as the epidermal growth factor receptor (EGFR) and the insulin receptor, altering the balance of survival signals towards death [10].

The process of apoptosis is under the control of a variety of internal and external signals that activate the mitochondrial pathway or the death receptor pathway, respectively [22,23]. Members of the multidomain Bcl-2 gene family play a key regulatory role in the mitochondrial pathway by eithersuppressing or promoting apoptosis. The antiapoptotic members include Bcl-2, Bcl-XL, Bfl-1, Bcl-W, and Mcl-1, whereas the proapoptotic members include Bax, Bak, and Bik. Activated Bax/Bak induces apoptosis by causing outer mitochondrial membrane permeabilization and release of cytochrome c, leading to cleavage of caspase-9, caspase-3, and eventually poly(ADP-ribose) polymerase (PARP). The activation of Bax/Bak is blocked by Bcl-2/Bcl-XL that function as decoy receptors. Ultimately, it is the net balance between antiapoptotic and proapoptotic proteins in the cell that determines cell fate [22]. Activation of caspase-9, caspase-3 plays a central role in apoptosis by initiating cell death [24]. Caspase-3 has substrate specificity for the amino acid sequence Asp-Glu-Val-Asp (DEVD) and cleaves poly (ADP-ribose) polymerase (PARP). And activated caspase-3 is the key mediator of cell apoptosis cleaving intracellular proteins vital for cell survival and growth, such as PARP. It has been demonstrated that the proteolytic cleavage of PARP is a biochemical event during apoptosis [25,26]. In this study, dsWT1-319 decreased anti-apoptotic protein Bcl-2, increased proapoptotic protein Bak and activated caspase-3, leading to PARP cleavage and the induction of apoptosis in dsWT1-319 treated HepG2 cells.

In conclusion, this study demonstrates that dsWT1-319 induced apoptosis in human hepatocellular carcinoma HepG2 cells. This is mediated through up-regulation of Bak, down-regulation of Bcl-2, and activation of caspase-3 and PARP. The results of our study provide evidences that up-regulation of WT1 by dsWT1-319 may have therapeutic potential in the treatment of hepatocellular carcinoma

## Competing interests

The authors declare that they have no competing interests.

## Authors' contributions

QQ conducted the experiments, analyzed data and drafted the manuscript. SJH and ZYZ also analyzed the data and assisted with manuscript preparation. YWL, HC, QQM and KY assisted with experiments and manuscript preparation. XYZ revised the manuscript. All authors have read and approved the final manuscript.

## References

[B1] El-Serag HB Hepatocellular carcinoma: an epidemiologic view J Clin Gastroenterol 2002 35 5 Suppl 2 S72 78 12394209 10.1097/00004836-200211002-00002

[B2] Parkin DM Pisani P Ferlay J Global cancer statistics CA Cancer J Clin 1999 49 1 33 64 31 10.3322/canjclin.49.1.33 10200776

[B3] Bruix J Llovet JM Prognostic prediction and treatment strategy in hepatocellular carcinoma Hepatology 2002 35 3 519 524 10.1053/jhep.2002.32089 11870363

[B4] Li LC Okino ST Zhao H Pookot D Place RF Urakami S Enokida H Dahiya R Small dsRNAs induce transcriptional activation in human cells Proc Natl Acad Sci USA 2006 103 46 17337 17342 10.1073/pnas.0607015103 17085592 PMC1859931

[B5] Janowski BA Younger ST Hardy DB Ram R Huffman KE Corey DR Activating gene expression in mammalian cells with promoter-targeted duplex RNAs Nat Chem Biol 2007 3 3 166 173 10.1038/nchembio860 17259978

[B6] Wang J Place RF Huang V Wang X Noonan EJ Magyar CE Huang J Li LC Prognostic value and function of KLF4 in prostate cancer: RNAa and vector-mediated overexpression identify KLF4 as an inhibitor of tumor cell growth and migration Cancer Res 2010 70 24 10182 10191 10.1158/0008-5472.CAN-10-2414 21159640 PMC3076047

[B7] Yang L Han Y Suarez Saiz F Minden MD A tumor suppressor and oncogene: the WT1 story Leukemia 2007 21 5 868 876 17361230 10.1038/sj.leu.2404624

[B8] Hohenstein P Hastie ND The many facets of the Wilms' tumour gene, WT1 Hum Mol Genet 2006 15 Spec No 2 R196 201 16987884 10.1093/hmg/ddl196

[B9] Tatsumi N Oji Y Tsuji N Tsuda A Higashio M Aoyagi S Fukuda I Ito K Nakamura J Takashima S Wilms' tumor gene WT1-shRNA as a potent apoptosis-inducing agent for solid tumors Int J Oncol 2008 32 3 701 711 18292948

[B10] Menke AL Shvarts A Riteco N van Ham RC van der Eb AJ Jochemsen AG Wilms' tumor 1-KTS isoforms induce p53-independent apoptosis that can be partially rescued by expression of the epidermal growth factor receptor or the insulin receptor Cancer Res 1997 57 7 1353 1363 9102224

[B11] Perugorria MJ Castillo J Latasa MU Goni S Segura V Sangro B Prieto J Avila MA Berasain C Wilms' tumor 1 gene expression in hepatocellular carcinoma promotes cell dedifferentiation and resistance to chemotherapy Cancer Res 2009 69 4 1358 1367 10.1158/0008-5472.CAN-08-2545 19190340

[B12] Huang V Qin Y Wang J Wang X Place RF Lin G Lue TF Li LC RNAa is conserved in mammalian cells PLoS One 2010 5 1 e8848 10.1371/journal.pone.0008848 20107511 PMC2809750

[B13] Place RF Li LC Pookot D Noonan EJ Dahiya R MicroRNA-373 induces expression of genes with complementary promoter sequences Proc Natl Acad Sci USA 2008 105 5 1608 1613 10.1073/pnas.0707594105 18227514 PMC2234192

[B14] Kuwabara T Hsieh J Nakashima K Taira K Gage FH A small modulatory dsRNA specifies the fate of adult neural stem cells Cell 2004 116 6 779 793 10.1016/S0092-8674(04)00248-X 15035981

[B15] Morris KV Santoso S Turner AM Pastori C Hawkins PG Bidirectional transcription directs both transcriptional gene activation and suppression in human cells PLoS Genet 2008 4 11 e1000258 10.1371/journal.pgen.1000258 19008947 PMC2576438

[B16] Schwartz JC Younger ST Nguyen NB Hardy DB Monia BP Corey DR Janowski BA Antisense transcripts are targets for activating small RNAs Nat Struct Mol Biol 2008 15 8 842 848 10.1038/nsmb.1444 18604220 PMC2574822

[B17] Clark AJ Archibald AL McClenaghan M Simons JP Wallace R Whitelaw CB Enhancing the efficiency of transgene expression Philos Trans R Soc Lond B Biol Sci 1993 339 1288 225 232 10.1098/rstb.1993.0020 8097052

[B18] Hartkamp J Carpenter B Roberts SG The Wilms' tumor suppressor protein WT1 is processed by the serine protease HtrA2/Omi Mol Cell 2010 37 2 159 171 10.1016/j.molcel.2009.12.023 20122399 PMC2815029

[B19] McMaster ML Gessler M Stanbridge EJ Weissman BE WT1 expression alters tumorigenicity of the G401 kidney-derived cell line Cell Growth Differ 1995 6 12 1609 1617 9019166

[B20] Englert C Hou X Maheswaran S Bennett P Ngwu C Re GG Garvin AJ Rosner MR Haber DA WT1 suppresses synthesis of the epidermal growth factor receptor and induces apoptosis EMBO J 1995 14 19 4662 4675 7588596 10.1002/j.1460-2075.1995.tb00148.x PMC394563

[B21] Sera T Hiasa Y Mashiba T Tokumoto Y Hirooka M Konishi I Matsuura B Michitaka K Udaka K Onji M Wilms' tumour 1 gene expression is increased in hepatocellular carcinoma and associated with poor prognosis Eur J Cancer 2008 44 4 600 608 10.1016/j.ejca.2008.01.008 18255279

[B22] Morrison DJ English MA Licht JD WT1 induces apoptosis through transcriptional regulation of the proapoptotic Bcl-2 family member Bak Cancer Res 2005 65 18 8174 8182 10.1158/0008-5472.CAN-04-3657 16166292

[B23] Bouillet P Strasser A BH3-only proteins - evolutionarily conserved proapoptotic Bcl-2 family members essential for initiating programmed cell death J Cell Sci 2002 115 Pt 8 1567 1574 11950875 10.1242/jcs.115.8.1567

[B24] Salvesen GS Dixit VM Caspase activation: the induced-proximity model Proc Natl Acad Sci USA 1999 96 20 10964 10967 10.1073/pnas.96.20.10964 10500109 PMC34227

[B25] Germain M Affar EB D'Amours D Dixit VM Salvesen GS Poirier GG Cleavage of automodified poly(ADP-ribose) polymerase during apoptosis. Evidence for involvement of caspase-7 J Biol Chem 1999 274 40 28379 28384 10.1074/jbc.274.40.28379 10497198

[B26] Ivana Scovassi A Diederich M Modulation of poly(ADP-ribosylation) in apoptotic cells Biochem Pharmacol 2004 68 6 1041 1047 10.1016/j.bcp.2004.04.023 15313399

